# The HIV-1 pandemic: does the selective sweep in chimpanzees mirror humankind’s future?

**DOI:** 10.1186/1742-4690-10-53

**Published:** 2013-05-24

**Authors:** Natasja G de Groot, Ronald E Bontrop

**Affiliations:** 1 Department of Comparative Genetics and Refinement, Biomedical Primate Research Centre, Lange Kleiweg 161, 2288 GJ Rijswijk, The Netherlands; 2 Department of Theoretical Biology and Bioinformatics, Utrecht University, 3584 CH Utrecht, The Netherlands

**Keywords:** AIDS, Chimpanzee, HIV-1, HLA, Human, MHC, Repertoire reduction, SIV_cpz_, Zoonosis

## Abstract

An HIV-1 infection progresses in most human individuals sooner or later into AIDS, a devastating disease that kills more than a million people worldwide on an annual basis. Nonetheless, certain HIV-1-infected persons appear to act as long-term non-progressors, and elite control is associated with the presence of particular MHC class I allotypes such as HLA-B*27 or -B*57. The HIV-1 pandemic in humans arose from the cross-species transmission of SIV_cpz_ originating from chimpanzees. Chimpanzees, however, appear to be relatively resistant to developing AIDS after HIV-1/SIV_cpz_ infection. Mounting evidence illustrates that, in the distant past, chimpanzees experienced a selective sweep resulting in a severe reduction of their MHC class I repertoire. This was most likely caused by an HIV-1/SIV-like retrovirus, suggesting that chimpanzees may have experienced long-lasting host-virus relationships with SIV-like viruses. Hence, if natural selection is allowed to follow its course, prospects for the human population may look grim, thus underscoring the desperate need for an effective vaccine.

## Review

### AIDS: facts and figures

Acquired immunodeficiency syndrome (AIDS) is caused by the human immunodeficiency virus type 1 (HIV-1) and poses a major threat to human health. At present, over thirty million individuals are infected worldwide, and more than twenty five million people have died from AIDS since the beginning of the pandemic (UNAIDS 2011). Based on genetic characteristics, the virus strains can be clustered into different groups designated M, N, O and P. HIV-1 group M is responsible for the vast majority of infections recorded in human individuals globally [1,2]. Thanks to the currently available anti-retroviral (AR) drugs, the number of people dying yearly from AIDS reached a plateau around 2004, and the number has even begun to decline (UNAIDS 2011). However, it is important to mention that highly active anti-retroviral therapy (HAART) does not eradicate HIV-1 [3], and several studies have demonstrated that discontinuation of HAART may result in a rapid viral rebound [4-7]. Moreover, AR therapy carries with it high costs, resulting in only a limited number of HIV-1-infected individuals having access to it. Especially in developing countries, where most of the infected individuals live, AIDS is still a prominent health problem. Another worry is that sooner or later the virus may develop resistance to these drugs [8], and the spread of drug-resistant HIV-1 strains would be devastating. Therefore, a series of low-cost drugs and, more optimistically, an efficacious vaccine able to protect against infection are urgently needed.

### HIV-1 in a nutshell: its genome and infection routes

**Figure 1:**
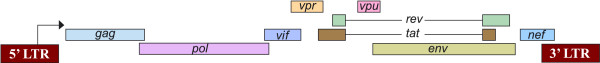
**Schematic representation of the HIV-1 genome and its gene products.** The arrow indicates the transcription initiation site.

The genome of HIV-1 is relatively small, approximately 10 kilobases, and encodes a limited number of gene products (Figure 1). This retrovirus belongs to the group of lentiviruses. Like most retroviruses, the HIV-1 genome contains the three functional proteins Gag, Pol, and Env, which are essential for the construction of the virus particle [9]. In addition, lentiviral genomes encode a number of regulatory (Tat and Rev) and accessory genes (Vif, Vpr, Vpu, and Nef). The Tat and Rev proteins are essential for replication of the virus. The accessory proteins are attached to viral messenger RNA, but they are not required for replication of the virus *in vitro*. However, *in vivo* these proteins are necessary for the replication and virulence of the virus [10].

HIV-1 possesses specific characteristics that hamper or have hampered the development of broadly efficacious and efficient anti-retroviral drugs and vaccines. On the one hand, the virus can cause a persistent infection and hide in an inactive form in cellular reservoirs. On the other hand, it can replicate quickly, and − depending on the copy number present in one individual − may produce 10^9^ to 10^10^ particles per day [11,12]. Moreover, the genome of HIV-1 encodes an error-prone reverse transcriptase characterized by a high mutation rate (approximately 3 × 10^-5^ per nucleotide/replication cycle) [13]. This, in combination with a relatively small genome size and the high viral production rate within a given individual, may ultimately result in the establishment of a virus swarm. There is also the risk of emergence of new recombinant strains as multiple HIV-1 viruses, which are prone to exchange genetic material, infect a single cell. Hence, a high mutation rate in concert with recombination provides the biological ability for HIV-1 to escape the immune recognition/control of its host.

HIV-1 enters the human body via mucosal sites, blood-blood contact, or breast milk [14]. After entry, the virus either directly infects CD4^+^ T cells, is recruited by dendritic cells (DC), or is taken up by macrophages. The latter two cell types act as a kind of Trojan horses, and transport HIV-1 through different layers of epithelial cells to the lymphoid tissues, where they can transfer the virus to CD4^+^ T cells [15,16]. DCs can either internalize HIV-1 into endosomes, and the cell-surface molecule that is used by the virus for entry into the cell is the C-type lectin receptor DC-SIGN [17], or they can recruit HIV-1 virions to their endosomal compartment, where the virions accumulate at regions that come into contact with neighboring T cells (referred to as the infectious synapse) [18]. Macrophages may phagocytize HIV-1, and can either function as a reservoir for long-term persistence of the virus or can be responsible for the transport and dissemination of the virus. The “primary” non-syncytium-inducing (NSI) HIV-1 viruses mainly infect CD4^+^ memory T cells that express CC-chemokine receptor-5 (CCR5). Later in the course of the infection, the NSI strains can switch to a syncytium-inducing (SI) phenotype, which can infect T cells by using the C-X-C-chemokine receptor-4 (CXCR4) as co-receptor. This switch is associated with a loss of sensitivity to chemokines (like RANTES, and MIP-1α and β), a rapid decrease in CD4^+^ T cells, and progression towards AIDS [19]. Moreover, an infection of memory CD4^+^ T cells that revert back to a resting state ensues as well, resulting in a dormant stage of the virus that is undetectable by the host’s immune system [20]. In essence, HIV-1 infection and its subsequent manipulations take place at the heart of the immune system.

### Zoonotic infections: what may happen if we open Pandora’s box

More than 40 African non-human primate species are infected naturally with various simian immunodeficiency virus (SIV) strains, but progression towards AIDS is rarely observed [21,22]. From among these natural primate hosts, the SIV infection has been most thoroughly studied in three species, sooty mangabeys, African green monkeys, and mandrills, and has led to the insight that non-pathogenicity need not be linked to effective immune control. Apparently, many of the species have co-evolved with SIV infections, and have found ways to manage/control it. These non-human primates, however, are potential reservoirs for viruses that could be spread among related species. One example of such a zoonotic transmission is provided by sooty mangabeys (*Cercocebus atys*). This species, infected naturally with a SIV strain (SIV_sm_), is considered to represent the origin of the HIV-2 infection in humans, causing an AIDS-like disease [23]. The infection in humans is mostly confined to the West African territory, and indeed overlaps with the natural habitat of sooty mangabeys.

**Figure 2:**
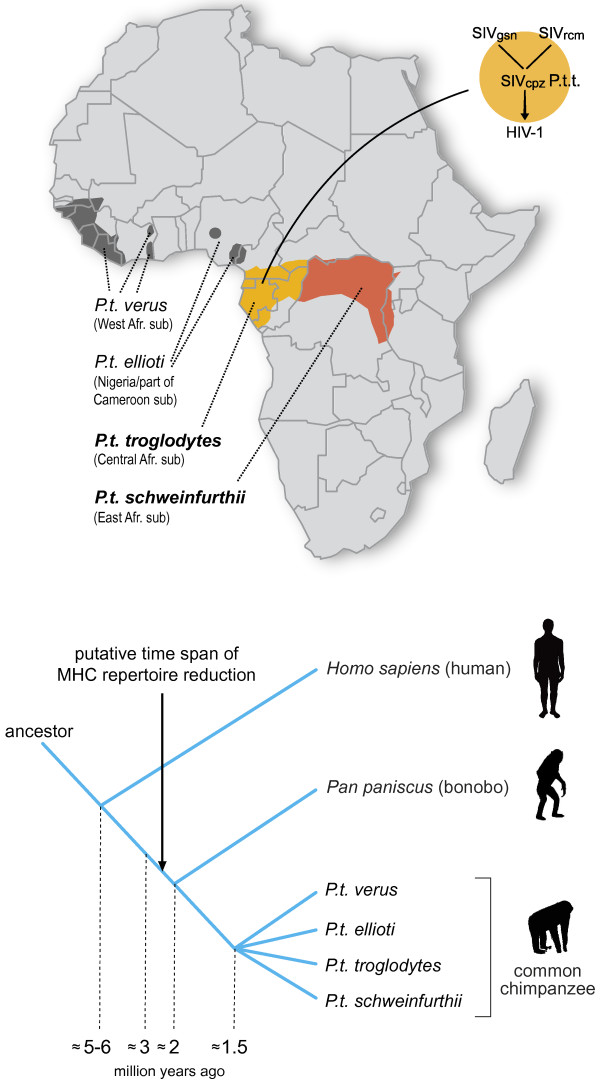
**Map of the African continent, highlighting habitats of the four different chimpanzee subspecies.** Populations that show evidence of contemporary natural infections with SIV_cpz_ strains are *Pan troglodytes troglodytes* (light orange) and *P.t. schweinfurthii* (dark orange). Superimposed is a diagram illustrating the cross-species transmission events that led to the emergence of HIV-1 group M, the initiator of the human pandemic [28]. The lower panel in the figure illustrates the speciation events in the homo-pan lineage. The arrow indicates the putative time span of the MHC repertoire reduction in the pan lineage.

Chimpanzees (*Pan troglodytes*), humankind’s closest living relative, can also be infected with SIV [24]. Based on the geographic distribution as well as on morphological and genetic data, chimpanzees have been divided into four different subspecies/populations sharing a common ancestor approximately 1.5 million years ago [25-27], and incidences of natural SIV_cpz_ infection have been recorded in contemporary animals from at least two of the four subspecies (Figure 2). These SIV_cpz_-strains appear to have a mosaic genome, consisting of gene segments from different types of SIV strains obtained from distinct Old World monkey species [28]. This suggests that chimpanzees have acquired their types of SIV infections by predation on Old World monkeys sharing the same habitat. In turn, the different HIV-1 groups M, N, O and P, have arisen from at least four separate introductions of SIV into the human population. The initiator of the human pandemic (HIV-1 group M) is the SIV_cpz_ strain derived from chimpanzees of the Central-African subspecies (*Pan troglodytes troglodytes*) [29] (Figure 2), and the HIV-1 group N also arose from an SIV_cpz_ strain from this species. The HIV-1 groups O and P most likely originated from gorillas (SIV_gor_) [30,31], in which there is an indication that *P.t.t.* animals are the source of SIV_gor_[32].

### Chimpanzees and AIDS

Apart from humans, chimpanzees are one of the non-human primate species that is susceptible to infection with HIV-1. Recently, it has been shown that pig-tailed macaques can also be infected through a modified HIV-1 strain that only differs in the *vif* gene [33,34]. In the past, a substantial number of West-African chimpanzees (*P.t.verus*) were experimentally infected with different HIV-1 strains, but most of these animals seem to be relatively resistant to developing AIDS [35,36]. These experiments were conducted before it was known that HIV-1 was derived from a zoonotic transmission of a virus that has its roots in a chimpanzee reservoir. In the late 1980s and early 1990s, the first chimpanzees to be infected naturally with an HIV-1-related lentivirus were discovered [37-39]. Additional natural SIV_cpz_ infections in captive and wild-living chimpanzees were described later [40,41]. Some of these chimpanzees were kept under close surveillance for long periods, but signs of an AIDS-like disease were not seen. Moreover, transmission of one of the viruses to West-African chimpanzees (animals in which natural infection with SIV_cpz_ is not observed) also did not result in the progression to AIDS [42]. Recently, population analyses have shown that wild-ranging East-African chimpanzees (*P.t. schweinfurthii*) experience natural infections with SIV_cpz_. For one of the populations, evidence of AIDS-like symptoms has been documented [43], and AIDS-related symptoms have also been described for a naturally infected Central-African chimpanzee [44]. In addition to these observations of AIDS in SIV_cpz_-related infections, there is one case documenting that chimpanzees can develop AIDS after experimental infection with HIV-1 [45]. This chimpanzee of West-African origin was infected with three different HIV-1 isolates. The animal eventually developed AIDS, and subsequent analysis showed that a recombinant HIV-1 strain had emerged. Three other chimpanzees that were afterwards infected with this recombinant strain all developed signs of AIDS-like disease [46]. This set of experiments shows that viruses that have been edited in the human population, and that are re-introduced into their original host species, can cause AIDS. Thus, escape from immune control in chimpanzees is possible in exceptional cases, even within a species considered to be relatively resistant.

### Control of AIDS development in humans: an introduction to the MHC

In HIV-1-infected human cohorts, certain individuals do not progress towards AIDS. Some of these long-term non-progressors (LTNP)/elite controllers (ECs) have been infected for more than two decades [47], and their situation appears to resemble that of HIV-1-infected chimpanzees in terms of their relative resistance to developing AIDS. The LTNP/EC status in humans is strongly associated with the presence of particular major histocompatibility complex (MHC) class I molecules, such as HLA-B*27:05 and -B*57:01 [48-54].

The MHC plays a central role in the induction of adaptive immune responses, and encodes two clusters of cell-surface proteins. In humans, these molecules are designated HLA-A, -B, and -C (class I cluster), and -DP, -DQ, and -DR (class II cluster), and almost all these genes display abundant levels of polymorphism [55]. MHC class I antigens are expressed on virtually all nucleated cells, and generally present peptides of 8-10 amino acids in length. One of the biological functions of MHC class I molecules is to sample degraded peptide fragments from pathogens that are able to establish intracellular infections. In most cases, such peptides are derived from viruses trying to manipulate the intracellular machinery of the host cell in favor of their own replication purposes. MHC class II molecules, on the other hand, display differential tissue distribution and usually bind substantially longer peptides. They sample degraded peptide fragments from pathogens that are establishing extra-cellular infections, or that have life stages that are taking place outside of the host cell. In the case of an intracellular infection, the immune system will try to kill the target cell by inducing apoptosis-mediated lysis by CD8^+^ cytotoxic T cells (CTL). Extra-cellular infections are usually eliminated by antibodies that are generated by B cells under the control of CD4^+^ T helper cells.

In order to initiate an immune response, each individual will present a range of antigenic peptides as dictated by their unique HLA repertoire. Thus, MHC polymorphism warrants that many individuals within a given population have the capacity to generate unique adaptive immune responses to eliminate infections. In other words, MHC polymorphism minimizes the chance that a given pathogen will eliminate an entire population. Several pathogens have evolved different ways to interfere with the MHC class I and II antigen presentation pathways in order to avoid immune recognition [56-59]. Indeed, it has been suggested that in human populations exposed to HIV-1 there is enrichment for particular HLA specificities [60,61].

### The MHC and resistance to the development of AIDS in chimpanzees

*Grosso modo*, humans and chimpanzees share the same MHC class I and II loci. However, chimpanzees possess an additional oligomorphic locus designated *Patr-AL* (*A-like*), which is characterized by a differential haplotype distribution and low expression levels [62,63]. An overview of the reported number of alleles for each locus is provided (Table 1). With regard to lineages, in comparison to humans, chimpanzees have a reduced MHC class I repertoire. The very first indications that chimpanzees may lack the evolutionary equivalents of particular *HLA* lineages became evident in serological studies [64]. In humans, the *HLA-A* locus alleles are divided into two lineages comprising six families (Table 2) [65,66], and the experiments of van Rood *et al.*[64] showed that chimpanzees lack the HLA-A2 related serotypes. Subsequent molecular studies suggested that chimpanzees might only possess the equivalents of the *HLA-A1/A3/A11/A30* family [67]. Although the number of animals tested in this initial study was relatively small, the outcome was unexpected. Humans and chimpanzees are each other’s closest living relatives, and they shared a common ancestor about 5 million years ago [68]. Thus, the most logical explanation would be that humans and chimpanzees share for the *A* and *B* loci the same lineages, since they were inherited in a trans-species mode of evolution [69].

**Table 1:** **Number of alleles detected for *Mhc *class I and II loci in humans (*HLA*) and chimpanzees (*Patr*)**

	**Locus**	***HLA***	***Patr***
	*A*	2188	33
***Mhc class I***	*B*	2862	57
	*C*	1746	31
	*E*	11	2
	*F*	22	1
	*G*	50	1
	*AL*	0	5
	*DPA1*	36	3
	*DPB1*	159	29
***Mhc class II***	*DQA1*	49	7
	*DQB1*	193	10
	*DRA*	7	2
	*DRB*	1386	79
**#Individuals analyzed**		>>	≈100

Data were retrieved from the databases http://www.ebi.ac.uk/imgt/hla[70] and http://www.ebi.ac.uk/ipd/mhc/nhp[71].

**Table 2:** **Subdivision of the *HLA-A *locus members into lineages and families**

**Lineage**	**Family**
	*HLA-A2*
**A2**	*HLA-A10*
	*HLA-A19*
	*HLA-A1/A3/A11/A30*
**A3**	*HLA-A9*
	*HLA-A80*

**Figure 3:**
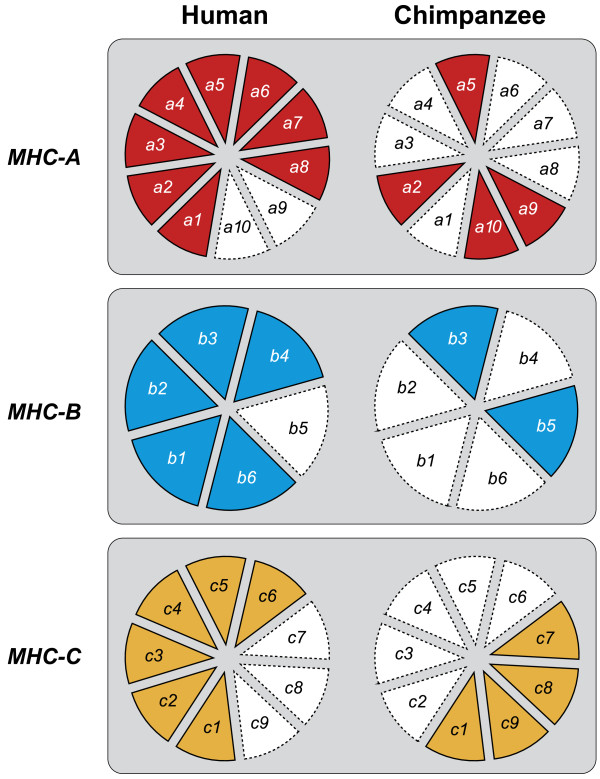
**Pie charts showing the presence/absence of MHC class I intron 2 lineages in humans and chimpanzees.** A colored section in a pie indicates the presence of a particular intron 2 lineage in that species; red for *MHC-A*, blue for *MHC-B*, and orange for *MHC-C*.

**Figure 4:**
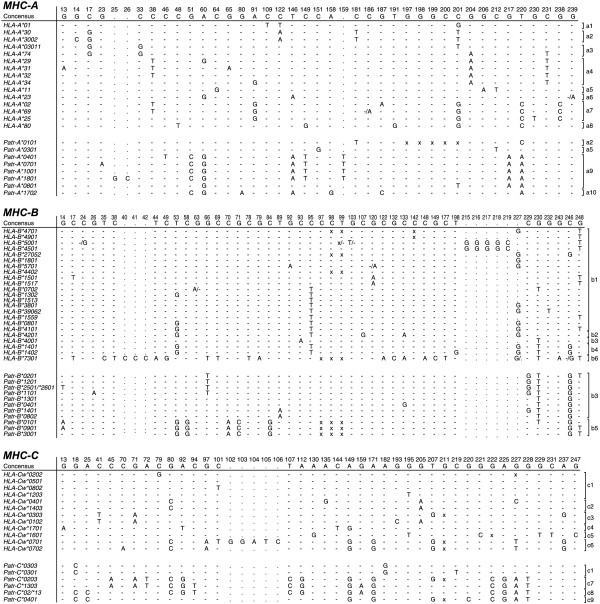
**MHC class I intron 2 sequences in humans (*HLA*) and chimpanzees (*Patr*).** Only the polymorphic nucleotide positions are indicated. Identity to the consensus sequence (depicted at the top) is indicated by dashes. Substitutions and inserts are depicted by the conventional one-letter code; deletions are marked “x”. For instance, “-/A” indicates that differences in a particular sequence have been reported in the literature. The brackets indicate the division of the intron 2 alleles into lineages, and this is based on phylogenetic analysis. (The figure is adapted from De Groot N.G. et al., PNAS 99, 11748-11753, 2002).

As a follow-up to McAdam’s study [67], the MHC class I repertoire of a pedigreed West-African chimpanzee colony comprising more than 30 wild-caught founder animals was analyzed. Again, only orthologs of the *HLA-A1/A3/A11/A30* family were encountered [72]. Moreover, MHC analyses of West-African chimpanzees by other research teams, and of small numbers of Central- and East-African chimpanzees, also provided data indicating only the presence of *HLA-A1/A3/A11/A30* family orthologs [72-76]. Although the results suggest that there was a repertoire reduction at the MHC class I region in chimpanzees, conclusions in this direction should be arrived at cautiously. The presence of one particular omnipresent *HLA-A*-like family in chimpanzees can, in theory, also be explained by convergent evolution. Additionally, the imbalance in sample size between the number of chimpanzees and humans analyzed for their MHC repertoire can hamper an accurate interpretation of the data. For instance, one can argue that particular alleles and/or lineages were missed in chimpanzees due to a limited sample size. In addition, the MHC class I and II gene products of different species/populations may experience different sorts of natural selection, depending on their habitat and on the pathogens that are present. Another viable explanation could be that there was an expansion of the HLA class I lineages. To answer such questions, we designed a study that investigated the influence of selection operating on the classical MHC class I loci. This involved the comparison of intron variation of chimpanzee and human MHC class I alleles. The different intron 2 variations observed in both species are clustering into various lineages, of which some are shared (Figures 3 and 4). On the basis of two well-defined populations, the MHC class I intron 2 data regarding twenty-five randomly chosen human Caucasian individuals [77] were compared with the data regarding our twenty-five *P.t.v*. chimpanzees. The χ^2^ statistics showed that the intron 2 variation found in humans is 2.56 times higher (confidence interval (CI) 95% is 0.87-7.55, P = 0.07) for the MHC-A locus, and 2.64 times higher (CI 95% is 1.20-5.82, P = 0.01) for the B locus [78]. The human cohort used, however, was not typed for the *HLA-C* locus, and therefore we have reanalyzed the data using a different human cohort that is typed at high-resolution level for *HLA-A, -B,* and *-C* (random panel IHB). To determine for all three loci whether the number of intron 2 alleles detected in humans is significantly higher as compared to that in chimpanzees, the difference in unique number of alleles (∆n_e_) was calculated by using bootstrap analyses. Additionally, for each locus, the ratio of the number of unique alleles in humans divided by the number of unique alleles in chimpanzees was calculated. The ∆n_e_ and the ratios of unique alleles were considered statistically significant if their confidence interval did not enclose 0 or 1, respectively. The analyses revealed that all three loci show a statistical significant difference for the ∆n_e_ and ratio in humans versus chimpanzees (Table 3). Thus, the approach confirms that chimpanzees indeed experienced a selective sweep targeting the MHC class I repertoire. The repertoire condensation was claimed to predate the (sub)speciation of chimpanzees, as the same intron lineages are shared between the different populations of chimpanzees, and was calibrated to have happened approximately 2-3 million years ago [78] (Figure 2). This suggests that in the distant past, ancestors of the contemporary chimpanzee populations must have been in close contact with each other, and that a plague may have affected the entire species. The fact that chimpanzees experienced a repertoire reduction in the MHC class I region, and the knowledge that most chimpanzees infected experimentally with HIV-1 are relatively resistant to developing AIDS, resulted in the hypothesis that the ancient repertoire reduction may have been caused by an HIV-1/SIV_cpz_-like or a closely related retrovirus [78].

**Table 3:** **Difference in the number of unique alleles (∆n**_**e **_**(95% CI)) and the ratio of unique alleles (95% CI) in humans as compared to chimpanzees for the different MHC class I loci**

**Locus**	**Δn**_**e **_**(95% CI)**	**Ratio (95% CI)**
***Mhc-A***	4.00 (2.00 - 7.00)	1.80 (1.33 - 2.40)
***Mhc-B***	5.00 (2.00 - 8.00)	1.63 (1.22 - 2.17)
***Mhc-C***	6.00 (3.00 - 8.00)	2.25 (1.60 - 3.67)

For the statistical analysis, 50 haplotypes were re-sampled 100,000 times and with replacement, separately for both species. The ∆n_e_ was calculated by subtracting the n_e_ of the chimpanzee from the n_e_ of the human, and the ratio of unique alleles in the two species was calculated by dividing the n_e_ of the human by the n_e_ of the chimpanzee. The median was defined as the 50,000^th^ value and the lower and upper confidence limits were defined as the 250^th^ and 99,750^th^ values emanating from the bootstrapping. Calculations were performed using the R statistical package (version 2.15.3).

A successive comparative genomic approach, using microsatellite markers located in the MHC region and markers mapping at a variety of other chromosomes, revealed that both chimpanzees and humans show similar amounts of variation for most of the non-MHC markers. Nonetheless, a significant lower allelic variation for the majority of the markers mapping in the chimpanzee MHC region was documented [79]. These data support the claim that chimpanzees possess a reduced MHC repertoire. Subsequent multi-locus demographic analyses highlighted the fact that chimpanzees experienced a selective sweep that mainly targeted the chromosomal segment carrying the MHC class I region [79].

### The evolution of chimpanzee MIC genes: further substantiation of selection

Activated CD4^+^ memory T cells are selectively and rapidly depleted after an HIV-1/SIV infection [80,81]. This cell type is present in abundance in the intestine, but other mucosal surfaces (lung, vagina) that are frequently exposed to environmental pathogens also contain a high percentage of activated lymphocytes [82]. In humans and macaques in particular, the intestine is the prominent site of CD4^+^ T-cell depletion during the first few weeks after exposure to HIV-1/SIV [81,83]. The major histocompatibility complex class I chain-related gene (*MIC*) molecules are highly expressed on the gastrointestinal epithelium [84], and the genes encoding these molecules map to chromosome 6 within the MHC region. In humans, seven genes (*MICA* to *MICG*) have been distinguished, though only the *MICA* and *-B* genes produce functional transcripts and display high levels of polymorphism [70]. The molecules are thought to be involved in signaling stress to the immune system.

Chimpanzees appear to have only one functional *MIC* gene (localized near the *Patr-B* locus), which has an intermediate character resembling in part the human *MICA* and *-B* genes. A large ancestral deletion of 95 kb resulted in the birth of a hybrid *Patr-MICA/B* gene [85]. We have investigated *MIC* gene polymorphism in chimpanzees, and found that the *Patr-MICA/B* fusion gene controls only one lineage showing moderate allelic variation [86]. Thus, as compared to humans, chimpanzees have a reduced *MIC* gene repertoire, which is consistent with the selective sweep observed for the *Patr-B* locus. Moreover, the hybrid *MICA/B* gene appears to represent a fixed entity in all chimpanzee (sub)species. This is peculiar, as most other primate species possess haplotypes that carry both *MICA* and *-B* genes. Hence, somewhere during chimpanzee evolution a haplotype with a hybrid *MICA/B* gene was generated, and all other haplotypes carrying *MICA* and *-B* genes have gone missing. As the *MICA/B* gene maps next to the *Patr-B* gene, it is possible that selection on particular *B* alleles favored the preferential selection of this hybrid *MIC* gene by way of a piggyback effect. However, it cannot be excluded that the hybrid *MICA/B* gene itself, or the combination of this gene with particular *Patr-B* alleles, provided a selective advantage.

In humans, MIC is the ligand for the NKG2D receptor, which is expressed on natural killer (NK) cells, γδ T cells, and particular CD8^+^ αβ T cells [87]. A cellular “stress” signal, triggered for instance by a viral infection or malignant transformation, may upregulate the expression of MIC, which can ultimately lead to an immune response. The Patr-MICA/B fusion molecule is recognized by human Vδ1 γδ T cells specific for MICA and B, suggesting a conserved recognition site [88]. In addition, the NKG2D receptor of humans and chimpanzees is also highly similar [89]. Whether MIC plays a role in NK effector responses against HIV-1/SIV_cpz_ has yet to be proven, as does the functional role of the *Patr-MICA/B* gene in viral infections [86].

### Signs of selection beyond the MHC in chimpanzees

HIV-1 targets CD4^+^ T cells by the use of the CD4 receptor and different co-receptors. Orthologs of the human CD4 receptor, and of the CCR5 and CXCR4 co-receptors, are present in chimpanzees. In humans, several genetic CCR5 modifications are associated with resistance/susceptibility to HIV-1 infection [90]. The CCR5 variant that possesses a 32-base pair deletion (CCR5-∆32) conferring nearly complete resistance to HIV-1 infection in homozygous individuals is present in approximately 1% of the Caucasian population [91]. In chimpanzees, no 32-base pair deletion in the CCR5 gene has been observed, and the relative resistance of chimpanzees to developing AIDS after an HIV-1 infection cannot be attributed to this genetic modification [92-94]. Variations in the human 5′ *cis*-regulatory region of CCR5 (5′CCR5) were found to be associated with different transcription levels that influence HIV-1 entry and may affect disease progression [95]. Hence, the human 5′CCR5 haplotype that shows the lowest promoter activity resulting in control of AIDS development is the most common haplotype in chimpanzees [96]. By assuming a wide range of demographic histories, Wooding *et al.*[96] demonstrated that the human 5′CCR5 promotor region experienced balancing selection, and that, in contrast, the chimpanzee equivalent was affected by a selective sweep. This suggests that if the chimpanzee 5′CCR5 promotor region is linked to functional variants that influence progression to AIDS, it may contribute to the relative resistance of chimpanzees to developing AIDS after an HIV-1/SIV_cpz_ infection. Corresponding to this finding, MacFie *et al.*[97] demonstrated differential patterns of diversity for the HIV-related loci CCR5, CXCR4, and CX_3_CR1 in three chimpanzee subspecies (West- and Central-African, and Nigeria-Cameroon) [97]. For the CCR5 locus, they showed that it has low levels of diversity for all three subspecies, and this appeared to be tightly centered, as flanking loci displayed normal variation in all subspecies. The results suggest that the CCR5 locus experienced a selective sweep and that this may have predated subspeciation. For the Central-African chimpanzees, natural infections with SIV_cpz_ are documented [39], and a selective sweep at the CCR5 locus may be related to recent co-evolution with SIV_cpz_. For the other two subspecies, natural infections with SIV_cpz_ are not documented in the contemporary living animals [44,98]. The fact that these subspecies do show evidence for selection at CCR5 could indicate that they were infected with SIV_cpz_-like virus strains in the past, and that infections are rare or absent at present. Alternatively, the entire ancestral chimpanzee population may have been infected with an SIV_cpz_-like virus, resulting in a selection event at that stage for which the signature still can be measured. Depending on novel infections with SIV_cpz_ strains, particular selection forces are still operative.

Different cellular restriction factors, such as tripartite motif protein 5 alpha (TRIM5α) and apolipoprotein B mRNA-editing enzyme 3G (APOBEC3G), can target the intracellular replication of HIV-1 [99]. The APOBEC3G gene underwent strong positive selection, and during primate evolution this gene experienced several episodes of adaptations [100]. Polymorphisms in the human APOBEC3G gene have been documented, but its relationship with control of viral replication needs further investigation [101]. The chimpanzee APOBEC3DE, one of the other seven members of the APOBEC family, has potent antiretroviral activity against HIV-1. This is thought to be driven by an ancient lentiviral selective pressure dating back approximately 2 to 6 million years [102], which is consistent with our hypothesis that chimpanzees experienced a selective sweep caused by HIV-1/SIV_cpz_-like or a closely related retrovirus prior to chimpanzee (sub)speciation [78].

In addition, chimpanzees experienced selection on the fourth component (C4) gene of the complement system that maps in the MHC class III region [103]. Most humans possess two copies of the gene, designated C4A and C4B, and both genes are known to display size polymorphism due to the insertion of a complete endogenous retrovirus of 6.3 kb [104]. The long version of the C4A gene is found in humans, orangutans, and a variety of Old World monkeys [103,105]. However, chimpanzees only possess the equivalents of the short versions of the C4A and C4B genes. Since the presence of the long C4A gene predates human and Old World monkey speciation, the equivalent of this gene has most likely been lost during chimpanzee evolution.

Therefore, different research angles provide evidence that chimpanzees, as compared to humans, have experienced selection operating on the MHC and other genomic regions during their evolution. Chimpanzees shared a common ancestor with the bonobo (*Pan paniscus*) approximately 2 million years ago. A recent comparison of the bonobo and chimpanzee genome revealed that in chimpanzees, the MHC region in particular has experienced positive selection [106].

### Ancient selective sweep in chimpanzees most likely caused by an SIV-like retrovirus: supporting data from functional studies

**Figure 5:**
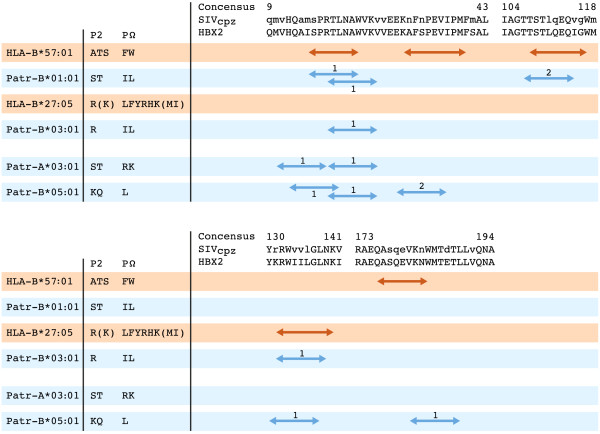
**Human (HLA) and chimpanzee (Patr) peptide-binding motifs of relevant MHC class I molecules, and the Gag protein regions that are potentially targeted.** P2 and PΩ (carboxyl-terminus) represent the anchor-binding positions of the MHC class I molecules; amino acids that are preferred on these positions are indicated by the conventional one-letter code, whereas tolerated amino acids are indicated between brackets. Patr-B*01:01 has a peptide-binding motif that resembles that of HLA-B*57:01, and Patr-B*03:01 has a peptide-binding motif that resembles that of HLA-B*27:05. Parts of the Gag consensus sequences of HIV-1 (HBX2) and SIV_cpz_ are given (http://www.hiv.lanl.gov). A lower-case letter in the SIV_cpz_ consensus indicates a variable position. The HLA-B*27 and -B*57 CTL epitopes are indicated by red arrows. For the respective Patr class I molecules, blue arrows indicate the potential Gag epitopes that are tested in peptide-binding studies. Based on IC_50_ (μM) values determined in peptide-binding competition assays, high (1) or intermediate (2) binding affinities of the peptides to their respective MHC class I molecule are indicated [109].

Control of AIDS development in HIV-1-infected human cohorts is strongly associated with the presence of HLA-B*27:05 and -B*57:01 [48,52-54,60], and adaptive immune responses to the HIV-1 Gag protein are thought to play an important role in the control of viral replication [107,108]. Hence, the Gag protein was taken as a model system to measure immune responses to this protein in chimpanzees. More specifically, we investigated whether the selective sweep had resulted in the preferential selection of Patr class I allotypes, and, if so, whether these allotypes could target similar HIV-1/SIV Gag regions as do HLA molecules associated with control of AIDS development. Therefore, the peptide-binding motifs of four Patr class I molecules, occurring at high frequency in a thoroughly characterized West-African chimpanzee population, were determined [109]. The obtained motifs were used to scan the HIV-1/SIV_cpz_ Gag proteins for potential CTL epitopes, and the relevant peptides were subsequently tested in binding studies. Two of the studied Patr molecules have peptide-binding motifs that resemble those of HLA-B*27 or -B*57, and can target similar areas of the HIV-1/SIV_cpz_ Gag protein (Figure 5). In addition, the two other studied allotypes, although divergent in their peptide-binding anchors, also appear to target the conserved areas of the HIV/SIV_cpz_ proteome similar to the AIDS-controlling HLA-B*27 and -B*57 molecules. Thus, particular human and chimpanzee allotypes share similar qualitative functional characteristics.

Recently, an in silico approach comparing human and chimpanzee MHC class I alleles illustrated that, with regard to the peptide-binding repertoire, the Patr-A molecules show signs of a selective sweep [110]. The Patr-A allotypes, in general, appear to have promiscuous binding profiles, and as a consequence they can target conserved areas of the Gag protein. The chimpanzee-specific Patr-AL molecule has a peptide-binding motif similar to HLA-A*02 [111]. However, as compared to the classical MHC class I loci, Patr-AL has distinct characteristics (low expression level and present only on approximately 50% of the chimpanzee MHC haplotypes), and further investigation is needed as to whether this molecule contributes significantly to adaptive immune responses.

In the founder population that we analyzed thoroughly, 94% of the chimpanzees possess at least one of the four Patr class I allotypes studied (Figure 5). Moreover, many chimpanzees were observed to express multiple allotypes, which are able to bind peptides derived from various conserved Gag regions. This has led us to suggest that chimpanzees may have developed a “double-lock” strategy to respond to an HIV-1/SIV_cpz_ infection, meaning that the chance of the virus escaping by mutations has been severely diminished. As only four Patr allotypes have been studied at this point, this quantitative aspect of the immune response might become more prominent if more peptide-binding motifs become available. Evaluation of the immune response data of three different HIV-1-infected chimpanzees revealed that the animals displayed broadly reactive CTL responses to conserved epitopes of the Gag protein [109,112,113]. Thus, the functional characteristics of the chimpanzee MHC class I repertoire suggest that the ancient selective sweep was caused by a lentiviral pandemic. The effect of this sweep can still be measured, as most extant chimpanzee populations appear to have a reduced MHC class I repertoire. However, most animals do possess (multiple) allotypes with functional characteristics similar to the AIDS-controlling HLA-B*27/B*57 molecules in humans.

There is evidence that the selective sweep or subsequent selection processes must have been more prominent in West-African chimpanzees than in other chimpanzee populations [96,97,114]. Population separation may have influenced this. Moreover, chimpanzees and their ancestors were most likely infected through predating on different monkey species that are infected with disparate types of SIV strains. Thus, depending on the monkey species predated upon, and their respective SIV infection, repertoires could have been edited in slightly different manners [109]. Predation may result in ongoing new infections. As a consequence, new recombinant SIV_cpz_ strains may be generated occasionally [115], and some of these strains may develop pathogenic characteristics. Thus, non-human primates are challenged by SIV infections for long periods of time, and must have developed ways to control the development of AIDS [22].

**Figure 6:**
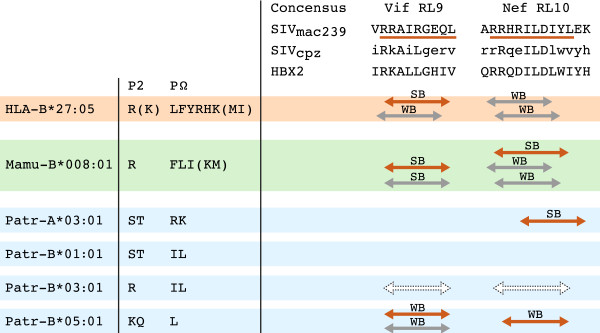
Conserved regions around the Mamu-B*008 epitopes Vif RL9 and Nef RL10 that can be targeted by HLA and Patr class I molecules. P2 and PΩ (carboxyl-terminus) represent the anchor-binding positions of the MHC class I molecules; amino acids that are preferred on these position are indicated by the conventional one-letter code, whereas amino acids indicated between brackets are tolerated. The epitopes Vif RL9 and Nef RL10 in SIV_mac239_ are indicated by a red line [118]. For HIV-1 (HBX2) and SIV_cpz_ the consensus sequences surrounding these Vif and Nef epitopes are given (http://www.hiv.lanl.gov). A lower-case letter in the SIV_cpz_ consensus indicates a variable position. A red arrow indicates that the respective MHC class I molecule is predicted to target the SIV_mac239_ consensus sequence. A grey arrow indicates prediction of the MHC molecule to target the SIV_cpz_/HBX2 consensus sequence. SB stands for solid binding (affinity < 100 nM); WB stands for weak binding (affinity between 100 to 700 nM). Binding affinity of the peptides was predicted using the NetMHCpan algorithm [119], except for Patr-B*03:01 (white dotted arrows), for which binding was assumed based on agreement in the anchor binding positions.

In rhesus macaque SIV models, currently used widely to study immunopathogenesis as well as to modulate the immunological responses induced by HIV-1 vaccine and vaccine components, EC is correlated with immune responses primarily directed towards the proteins Vif and Nef [116,117]. The macaque class I allotype Mamu-B*008, which is involved in EC, has a peptide-binding motif that resembles HLA-B*27, and only three CD8^+^ T-cell epitopes were shown to be responsible for the T-cell responses controlling replication of the pathogenic SIV_mac239_ in these ECs [118]. Two of the epitopes, Vif RL9 and Nef RL10, are conserved, and similar motifs are present in SIV_cpz_ and HIV-1 (Figure 6). Modeling the peptide binding of the proteins Vif and Nef to HLA-B*27:05 and the four studied Patr class I allotypes showed that they can target these conserved regions of Vif RL9 and Nef RL10 as well (Figure 6). This may suggest that, in different species, evolutionarily unrelated MHC class I molecules possessing similar peptide binding motifs are important for control of the lentiviral infection.

## Conclusions

### The selective sweep in chimpanzees: a mirror of humankind’s future?

A remaining question concerns how many chimpanzees died during the pandemic that may have been caused by an HIV-1/SIV_cpz_-like retrovirus. This is a difficult question to answer, but it is likely that the number must have been huge. Natural history has provided a few examples in which the introduction of a “novel” virus into a naïve population resulted in mass mortality. For instance, contact between European individuals and the native population of America led to the death of millions of Amerindians as a result of infection with measles, small pox, and other viruses [120]. The deliberate infection of rabbits with the myxoma virus exterminated approximately 99% of the rabbit population in Australia [121]. Natural infections in animals can also have strong effects on population size, as is illustrated by the rinderpest epidemic in African buffalo [122], and by the decimation of seals by morbilli and influenza viruses [123,124].

Modern humans, who have their cradle in Africa, have existed for about 150,000-200,000 years [125], and display an impressive amount of MHC class I polymorphism [126]. Recent cohort studies have demonstrated that particularly those individuals equipped with HLA-B*27 and/or -B*57 are able to control an HIV-1 infection [49,50]. Nevertheless, in these individuals one also sees that an escape by the virus may occur, although mostly with a cost to viral fitness [127-129]. Furthermore, there is an additional effect on viral control caused by a phenomenon such as heterozygous advantage [130,131]. Chimpanzees may reflect the latter stages of selection. First, the Patr molecules have the ability to respond to an HIV-1/SIV_cpz_ infection. Second, a quantitative aspect seem to be operative in chimpanzees, as each individual may possess several Patr molecules that can respond to different conserved parts of the HIV-1/SIV_cpz_ proteome, termed “double lock” strategy.

The selective sweep in chimpanzees resulted in the preferential selection of Patr class I allotypes that can target conserved areas of Gag similar to those targeted by HLA-B*27/B*57. In humans, these allotypes also seem to predispose for the immune-mediated pathologies ankylosing spondylitis and psoriasis, respectively. Evidence for the presence of these immune-mediated pathologies in chimpanzees is very low [132,133]. In humans, the exact role of HLA-B*27 in ankylosing spondylitis is also not known. Moreover, chimpanzee MHC class I molecules can target similar conserved motifs such as HLA-B*27/B*57, but this does not tell us whether the structure of the chimpanzee MHC class I molecules are comparable to HLA-B*27/B*57, as these molecules belong to different ancestral lineages [72,112].

In general, a virus survives best if it is able to replicate and disseminate itself within a population without killing all of its hosts. Such a host/virus state of equilibrium is, for instance, reached in different non-human primate species that are infected naturally with SIV strains. For HIV-1 and its human hosts, the battle seems to be in full swing, and without proper treatment the human population may be hit hard by this pandemic. For humans up until now, control of HIV-1 replication, without treatment, is significantly associated with the MHC region, in particular with the MHC class I molecules HLA-B*27:05 and -B*57:01, which can target conserved regions of the HIV-1 Gag protein. The observations adduced in this review have important consequences for vaccine design, as different HLA specificities may target different sections of the HIV-1 proteome. One major implication is that many human individuals may not possess HLA class I molecules that have the capacity to bind conserved HIV-1 epitopes that are associated with control of AIDS development. A truly protective vaccine has not yet been reported. Therefore, until that moment arrives, prevention of the infection itself will be, for the individual, one of the most important goals as regards surviving the current HIV-1 pandemic.

## Competing interests

The authors declare that they do not have competing interests.

## Authors’ contributions

NG and RB wrote the manuscript. Both authors have given their final approval of the manuscript.
